# Correlates of women's cancer screening and contraceptive knowledge among female emergency department patients

**DOI:** 10.1186/1472-6874-7-7

**Published:** 2007-05-22

**Authors:** Roland C Merchant, Erin M Gee, Beth C Bock, Bruce M Becker, Melissa A Clark

**Affiliations:** 1 Department of Emergency Medicine, Warren Alpert Medical School of Brown University, Providence, Rhode Island, USA; 2 Department of Community Health, Warren Alpert Medical School of Brown University, Providence, Rhode Island, USA; 3 Department of Psychiatry and Human Behavior, Warren Alpert Medical School of Brown University, Brown University, Providence, Rhode Island, USA

## Abstract

**Background:**

Lack of knowledge regarding preventive health services for women might impede campaigns to expand these services in the emergency department setting. For 18–55-year-old English-speaking women visiting an urban emergency department, we aimed to: (1) Ascertain their knowledge regarding the applicability, purpose, and recommended intervals of three women's cancer screening and three contraceptive methods; and (2) Determine if patient age, race/ethnicity, medical insurance status, and current or recent usage of these methods are associated with greater or lesser knowledge about them.

**Methods:**

Emergency department-based survey on recent or current usage and knowledge about Pap smears, breast self-examinations, mammograms, condoms, birth control, and emergency contraception. Analyses included calculation of summary statistics and creation of multivariable logistic regression models.

**Results:**

Of 1,100 patients eligible for the study, 69.9% agreed to participate. Most of the participants were < age 35, white, single (never married and no partner), Catholic, and had private medical insurance. Participant's recent or current usage of a particular cancer screening or contraceptive method varied by type of method: Pap smear within the past year (69.1%), breast self-exam within the past month (45.5%), mammogram within the past year (65.7% for women age 45–55), condom usage during every episode of sexual intercourse (15.4%), current usage of birth control pills (17.8%), and ever use of emergency contraception (9.3%). The participants correctly answered 87.9% of all survey questions about condoms, 82.5% about birth control pills, 78.5% about breast self-exams, 52.9% about Pap smears, 35.4% about mammograms, and 25.0% about emergency contraception. In multivariable logistic regression models, survey participants who had private medical insurance and those who recently or currently used a given screening or contraceptive method had a greater odds of correctly answering all questions about each cancer screening or contraceptive method.

**Conclusion:**

Although these female ED patients demonstrated strong knowledge on some women's cancer screening and contraceptive methods, there were several areas of knowledge deficit. Women without private medical insurance and those who have not used a particular cancer screening or contraceptive method demonstrated less knowledge. Reduced knowledge about women's cancer screening and contraceptive methods should be considered during clinical encounters and when instituting or evaluating emergency department-based initiatives that assess the need for these methods.

## Background

In the United States, there were 107.5 million patient visits to emergency departments (EDs) in 2001, of which approximately half were by women [1]. For many women, the ED is a common source for their medical care [2-4]. For some of these women, visiting the ED might be the way they access or receive referrals for preventive care. In 1996, the US Preventive Services Task Force of the US Department of Health and Human Services recommended that clinicians use patient visits as opportunities to provide preventive health services [5]. The Society for Academic Emergency Medicine Public Health and Education Task Force responded to this call and considered the evidence amassed thus far in support of instituting or expanding these services for the ED setting [6,7]. In many cases, especially for women's preventive health, there were few ED-based studies on the subject. As a result, definitive recommendations were not possible for several preventive health services for women, such as for women's cancer screening and contraception.

Studies that assess the extent to which female ED patients need cancer screening and contraceptive services would help inform policy makers about which services should be offered. However, an appreciation of what ED patients know about the cancer screening and contraceptive methods being offered is also required. If female ED patients do not have a working knowledge of what is being offered to them, they might be less likely to accept it and be less able to correctly answer questions about their need for it. As a result of knowledge deficits, a misunderstanding of the extent of the need for cancer screening and contraceptive services by women who visit EDs could occur and incorrect policy decisions might be made. By appreciating the extent of female ED patient's knowledge about women's cancer screening and contraceptive methods, identifying which women might have lesser knowledge, discovering which methods in particular women have lesser knowledge about, and understanding what aspects about these methods they have reduced knowledge about could direct future educational endeavors and interventions to encourage women to receive them, whether in the ED or in follow-up.

Patient knowledge about cancer screening and contraception during a clinical encounter is also crucial for ED clinicians to obtain an accurate medical history, to direct appropriate medical interventions, and to make recommendations for such services. Unfortunately for the patient and the health care provider, female patients' knowledge about the purpose of and their prior usage of women's cancer screening and contraception is often limited or incomplete [8-16]. For example, in a survey on Papanicolaou (Pap) smear knowledge, Ideström et al. observed that 95% of women surveyed believed they knew the purpose of Pap screening, but only 62% could correctly identify the type of cancer it tested [9]. In a 2004 study by Lyons et al. of women undergoing pelvic examinations in the ED, 74% mistakenly believed that they had undergone a Pap smear [8]. Of the patients interviewed, 81.5% believed that they knew the purpose of a Pap smear; however, only 26% of those correctly indicated a Pap smear is for cervical cancer. In several studies, a lack of utilization of women's cancer screening or contraceptive methods is associated with knowledge deficits about these methods, and vice versa [14,17-24]. For example, in a 2000 study of Korean-American women by Han et al., those with less knowledge about the purpose of clinical breast exams were significantly less likely to have had one [18]. In a study of socio-economically disadvantaged women in the South Bronx by Carter et al., low knowledge about women's cancer screening was associated with underutilization of Pap smears, breast self-exams, and clinical breast exams [19].

By delineating knowledge deficits about women's cancer screening and contraceptive methods according to the demographic and other factors in the population to which they serve, ED clinicians can identify groups of women who might benefit from expanded information and teaching. ED clinicians can also anticipate which patients might be less able to provide accurate histories of their usage of these methods. Studies in other settings have demonstrated that readily identifiable demographic factors, such as education [14,15,21,22,25-27], marital status [20,21,28], employment [21,26], type or status of insurance [27,29], race or ethnicity [29,30], and age [9,11,22,26,27] are associated with greater or lesser women's cancer screening and contraceptive knowledge, depending upon the population and method studied. ED clinicians who are conscious of potential indictors of reduced knowledge among certain groups of patients might be able to offer more in-depth teaching to improve patient utilization of women's cancer screening and contraceptive methods.

Therefore, the goals of this study were (1) to ascertain the knowledge of 18–55 year-old women being treated in an ED on the applicability, purpose, and recommended timing for three women's cancer screening methods: Pap smears, breast self-examinations, and mammograms; and three contraceptive methods: birth control, emergency contraception, and condoms; and (2) to determine if readily identifiable demographic factors (age, race/ethnicity, and insurance status) as well as usage of these methods are associated with greater or lesser knowledge about these women's cancer screening and contraceptive methods.

## Methods

### Study design

The study was a survey of 18–55-year-old, English-speaking, female ED patients using a self-administered, written, anonymous, multiple choice, single-best answer questionnaire that was developed by the study authors. The hospital institutional review board approved the study as exempt from review and written informed consent.

### Study setting

**Figure 1:**
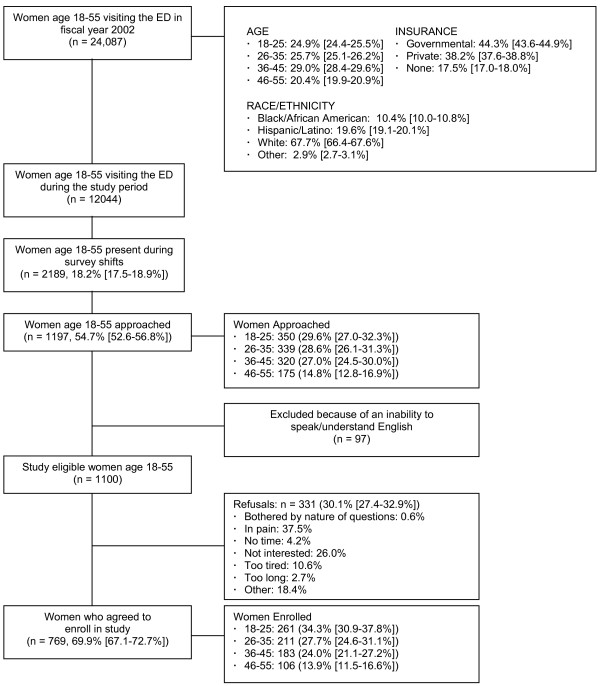
Enrollment diagram.

We conducted the study at an urban, Level I trauma, adult, academic, tertiary referral center, northeastern US ED with a catchment area of approximately 1.5 million people. In fiscal year 2002, there were 73,672 adult patient visits, and 49.0% of these were by women. Of these visits by women, 24,087 (66.7%) were by women between the ages of 18–55. Figure 1 shows the demographic profiles of these women.

### Survey design

We searched MEDLINE for surveys on women's health preventive topics and reviewed their contents, styles of questions, and survey methods. If a survey was not included in an article, we contacted the articles' authors and requested a copy. Fourteen surveys on various health topics were available for our review and four covered the topics in our study [28,31-43]. We chose six topics for our survey and created questions patterned on the styles used in the surveys from our review. These six topics were on women's cancer screening and contraceptive methods for which ED-based interventions might be possible---such as providing, making referrals for, or giving educational materials about these methods from the ED. The topics comprised three women's cancer screening methods: Pap smears, breast self-examinations, and mammograms; and three contraceptive methods: birth control, emergency contraception, and condoms. The survey included demographic and health history questions, as well as questions on the respondents' usage of and knowledge on these six methods. The cancer screening and contraceptive method knowledge questions asked the applicability, purpose, and recommended intervals for each of these methods. For example, the questions asked, "Who should have a Pap smear/Pap test?" (applicability), "Why do you think a Pap smear/Pap test is done?" (purpose), and "How often should most people go for a Pap smear/Pap test (unless otherwise directed by a doctor)?" (recommended intervals). A summary of the cancer screening and contraceptive knowledge questions are in Table three. The reading level of the entire survey was at a Flesch-Kincaid grade level of 5.4 (Microsoft Word, Redmond, WA).

We created a draft questionnaire and pilot tested it in June 2002 on a convenience sample of twenty female patients from the ambulatory care section of the ED. Using a standardized script, we interviewed these women after they completed the survey. The interview consisted of a brief cognitive assessment on randomly selected questions from the questionnaire, a review of potentially sensitive questions, and an analysis of mis-marked responses. We also solicited feedback on their opinions, impressions, and reactions to the questionnaire. Each of these women received $20 for participation in this pilot test. We revised the survey based upon their comments and our observations, e.g., poorly worded questions were rewritten, terms of confusion clarified, typographical errors corrected, etc. The final survey germane to this analysis included 36 multiple choice questions: six demographic questions, fourteen health history questions (cancer screening, sexual, contraceptive, and pregnancy history), and three knowledge-based questions on the applicability, purpose, and recommended intervals for each of the six topics (except emergency contraception, which had one question that assessed knowledge about the concept and purpose of emergency contraception).

### Survey administration

We chose to survey 18–55-year-old women, as we suspected that older women might have different health concerns (e.g. menopause) and were less likely to use the contraceptive methods mentioned in the survey. Women were excluded if they could not read or write in English; were being evaluated in the critical care, psychiatric section, or alcohol abuse holding areas of the ED; were not awake; or could not physically complete the form. Otherwise, any awake female patient present in the non-psychiatric care, non-alcohol abuse holding areas, and non-critical care area of the ED during the times data was collected was approached and asked to be in the survey if they met the age and English-speaking inclusion criteria. Patients were apprised of the nature, content, and purpose of the study and were asked to give their verbal consent to participate. Since this was the initial survey on this subject we had prepared, we limited the study to English-speaking patients as a pilot of the instrument for future studies that involve a more inclusive population of women. Each participant who completed the survey received a $2 gift card to a local pharmacy.

Research assistants and trained volunteers administered the survey in three two-month blocks that included 66 eight-hour shifts in July-August 2002, 32 four-hour shifts in October-November 2002, and 32 four-hour shifts in April-May 2003. Training of the research assistants and volunteers consisted of two-hour didactic sessions with the study authors on how to approach ED patients, which patients to approach, how to recognize exclusion criteria, and how to present patients with the self-administered survey. We purposely sampled three different seasons to avoid unknown time-dependent factors that might influence the pattern of ED visits. The data collection shifts were scheduled to reflect the time-dependent influx of patients to our ED. In brief, we structured our survey administration such that 25% of the shifts were between 7 a.m. and 11 a.m., 50% between 11 a.m. and 11 p.m., and 25% from 11 p.m. to 7 a.m. This ED's daily volume is essentially the same each day of the week. To reflect this pattern of patient visits, we conducted our survey so that each day of the week was represented equally.

### Data analysis

Two research assistants independently entered the data into separate Epi Info 2002 (Centers for Disease Control and Prevention, Atlanta, GA) databases. The research assistants completed a self-guided tutorial on Epi Info 2002 [44] and were trained by the primary author on how to review the data forms prior to conducting the data entry. The two independent databases were compared using the data compare feature of Epi Info 2002 for every entry. Errors were corrected to create a final database. We transferred the final database for analysis into Stata 8.2 (Stata Corporation, College Station, TX) using Stat/Transfer6 (Circle Systems, Seattle, WA). We tabulated the frequency of responses to each of the questions and calculated 95% confidence intervals for estimates when applicable.

For the logistic regression analyses, we created binary outcome variables for each of the women's cancer screening and contraceptive knowledge questions. These outcome variables were defined as correct vs. incorrect responses to each of the knowledge questions. Responses of "I am not really sure" were considered incorrect responses for this analysis. We further created a composite binary outcome variable that was composed of having responded correctly vs. incorrectly to all answers for a given topic. The analyses focused primarily upon these composite outcome variables. We conducted univariable logistic regression analyses using the log odds of the probability of correctly answering the questions as the outcome variable, and age group, race/ethnicity, private medical insurance, and recent/ever usage of a given cancer screening or contraceptive method as the independent variables. We then created multivariable logistic regression models using all of these independent variables. Independent variables were considered to be significantly related to the outcome at the α = 0.05 level of significance.

## Results

### Study enrollment and participant demographics

Of 1,197 women approached to participate in the survey, 97 were ineligible because of an inability to communicate in English. Of the remaining 1,100 women, 769 (69.9%) completed all or part of the questionnaire. These 769 participants comprised 35.1% of the women present in the ED when the survey was administered. The Figure shows the enrollment patterns for the study. Participation was slightly higher among the youngest age group. The majority of non-participating women reported pain as their primary reason for declining (37.5%); only a few were bothered by the description of the survey's content (0.6%). Participants who answered any part of the survey were included in this analysis. The number of responses to the questions decreased in relation to the length of the survey. Patients who did not complete the questionnaire had left the ED prior to its completion. The number of respondents for each question is provided throughout the tables.

Table 1 provides the respondent demographic profiles. Most of the women were under age 35, white, single (never married and no partner), Catholic, and had private medical insurance. Compared to all 18–55-year-old women visiting the ED in fiscal year 2002, the sample included slightly more 18–25-year-olds and slightly fewer 46–55-year-olds (Figure and Table 1). Compared to all 18–55-year-old women presenting to the ED in 2002, more respondents had private medical insurance. In comparison, fewer were white or Hispanic/Latina, about the same percentage were black, while more were of other racial/ethnic groups.

**Table 1:** Demographic profile of survey respondents

		**%**
	**AGE GROUP**	
n = 761	**18–25**	34.3
	**26–35**	27.7
	**36–45**	24.2
	**46–55**	13.9
		
	**RACIAL/ETHNIC SELF-IDENTITY**	
n = 754	**American Indian/Native American**	5.3
	**Asian/Pacific Islander**	1.9
	**Black/African-American**	12.1
	**Hispanic/Latina**	15.8
	**White**	58.2
	**Bi/Multiracial**	6.0
	**Other**	0.8
		
	**MARITAL/RELATIONSHIP STATUS**	
n = 767	**Single**	42.5
	**Married**	29.5
	**Divorced/Separated/Annulled**	14.3
	**Male partner**	11.0
	**Female Partner**	1.0
	**Widowed**	1.7
		
	**RELIGIOUS IDENTITY**	
n = 759	**Buddhist**	1.5
	**Catholic**	50.2
	**Hindu**	0.1
	**Jewish**	0.9
	**Muslim**	1.1
	**Protestant**	25.4
	**No religious preference**	16.9
	**Other**	4.0
		
	**HEALTH INSURANCE**	
n = 759	**Private**	50.9
	**Governmental**	32.8
	**No Insurance**	16.3

### Health history

Table 2 shows the responses to the cancer screening, sexual, contraceptive, and pregnancy history questions. Although a majority of women reported having a Pap smear within the past year (69.1%), fewer had examined their own breasts within the past month (45.5%), used any form of birth control (26.3%), always used condoms (16.8%) or ever used emergency contraception (9.3%). Among 45–55-year-old women who had not undergone a mastectomy, 65.7% had a mammogram within the prior year. (Participant's age group but not actual age was requested in the survey.) Among the entire sample, most women (74.3%) reporting having had intercourse with a man within the past month, and the majority of respondents (69.9%) had been pregnant at least once in their lifetime.

**Table 2:** Health history

**SCREENING HISTORY**							
		**n**			**%**		
**Last PAP smear (no hysterectomy)**		679					
Within past year					69.1		
>1 year ago					24.2		
Never					6.8		

**Last mammogram (all women, no mastectomy)**		731					
Within past year					22.9		
>1 year ago					14.5		
Never					6.27		

**Last mammogram (age 45-55, no mastectomy)**		102					
Within past year					65.7		
>1 year ago					29.4		
Never					4.9		

**Last BSE exam (no mastectomy)**		755					
Within past year					45.5		
>1 year ago					31.5		
Never					23.0		

**SEXUAL HISTORY**							
**Last time having sex with a man**		737					
< 1 week					48.2		
< 1 month					22.1		
< 6 months					9.9		
< 1 year					4.9		
> 1 year					9.5		
Never					5.4		
							
**Frequency of condom use**		739					
No intercourse/no sex with men					8.1		
Never					45.7		
Sometimes					17.1		
Most of the time					13.7		
Every time					15.4		
							
**CONTRACEPTIVE HISTORY**							
				**Yes**		**No**	
**Currently taking BCPs**		758		17.8		82.2	
**Using other BC**		749		11.6		88.4	
**Had a BTL**		750		25.6		72.6	
**Had a hysterectomy**		752		10.1		89.9	
**Use any form of BC**							
**Ever used EC**		752		9.3		90.7	
**Ever had an abortion**		748		23.7		76.3	
							
**PREGNANCY HISTORY**							
			**Yes**		**No**		**Not Sure**
**Currently pregnant**		759	26.0		93.0		4.6
**Ever pregnant**		760	69.9		30.1		
**Number of Times Pregnant**		758					
Never					29.0		
1					13.5		
2					17.3		
3					17.4		
4					12.1		
> 4					10.7		
							
KEY TO ABREVIATIONS							
PAP: Papanicolaou	BC(Ps): Birth Control (Pills)		EC: Emergency Contraception				
BSE: Breast Self Exam	BTL: Bilateral Tubal Ligation						

### Women's cancer screening and contraceptive knowledge questions

Table 3 provides a concise summary of each of the women's cancer screening and contraceptive knowledge questions and the percentage of women who answered the questions correctly. The questions answered correctly most often were about condom use and the question answered correctly the least often was about emergency contraception. For the five topics that had three questions each for that topic (who, why, and how often), the percentage of women who correctly answered all three questions for a given topic was lower than the percentage of women who correctly answered any one of the three questions for that topic. The percentage of women correctly answering the three questions within a topic was similar, except in three cases. Although most women understood the purpose of having a mammogram (86.7%), fewer could identify that "women over age 40 and/or those women instructed by their doctor" should have a mammogram (50.3%) or that mammograms are typically scheduled every five years (62.1%). Likewise, compared to other questions within their respective topics, fewer women could identify that breast self-examinations are recommended monthly (82.5%) and fewer understood that birth control pills are taken only by women (87.2%).

**Table 3:** Respondents answering cancer screening and contraceptive knowledge questions correctly

		**n**	**% correct**	**95% CI**
**PAP SMEAR**	**CORRECT ANSWERS**			
Who should get a Pap smear?	Adult women	729	72.7	69.3–75.9
Why is a Pap smear performed?	Cervical cancer screening	724	73.2	69.8–76.4
How often do most people undergo a Pap smear?*	Yearly	724	74.0	70.0–77.2
**All correct answers**		717	52.9	49.1–56.6
				
**BREAST SELF-EXAM**				
Who should examine their own breasts?	Adult women	728	91.4	89.1–93.3
Why should a woman examine her own breasts?	Breast cancer screening	726	94.2	92.3–95.8
How often should a woman examine her own breasts?	Monthly	725	82.5	79.5–85.2
**All correct answers**		721	78.5	75.3–81.4
				
**MAMMOGRAM**				
Who should have a mammogram?	Age 40+/advised by doctor	722	50.3	46.7–54.0
Why should a woman have a mammogram?	Breast cancer screening	723	86.7	84.0–89.1
How often should a woman typically have a mammogram?*	Every 5 years	718	62.1	58.5–65.7
**All correct answers**		718	35.4	31.9–40.0
				
**BIRTH CONTROL PILLS**				
Who may take birth control pills?	Only women	717	87.2	84.5–89.5
Why might someone take birth control pills?	Prevent pregnancy	718	95.8	94.4–97.2
How often should someone take birth control pills?	Daily	717	91.9	90.0–93.8
**All correct answers**		713	82.5	79.5–85.2
				
**EMERGENCY CONTRACEPTION**				
Can birth control pills taken after sex prevent pregnancy?	Yes	713	25.0	21.8–28.3
				
**CONDOMS**				
Who should use condoms during sex?	Anyone having sex	709	92.0	89.7–93.6
Why should someone use condoms during sex?	Prevent pregnancy/infection	707	96.5	94.8–97.7
How often should someone use condoms?	Always with sex	706	92.9	90.8–94.7
**All correct answers**		700	87.9	85.2–90.1

*n.b*. All questions are paraphrased here for conciseness.

*"Unless otherwise directed by a doctor" stated for these questions

### Logistic regression analyses

Table 4 provides the results of the univariable logistic regression analyses that compared ability to answer the cancer screening and contraceptive knowledge questions correctly by age group, racial/ethnic group, recent or current use of a particular cancer screening or contraceptive method, and by whether or not the respondent had private medical insurance. In these analyses, older age was a statistically significant predictor of correctly answering all questions about Pap smears and mammograms. Racial/ethnic group was a statistically significant predictor of Pap smear and mammogram knowledge, and was marginally predictive of breast self-examination and birth control pill knowledge. For example, compared to white women, other race women had a 0.28, black women had a 0.31, and Hispanic/Latina women had a 0.27 lesser odds of being able to answer all Pap smear questions correctly. Having private medical insurance was a statistically significant predictor for all cancer screening and contraceptive knowledge questions. For example, women with private medical insurance had a 3.14 greater odds of answering all Pap smear questions correctly. Recent or current usage of a cancer screening or contraceptive method (ever usage for emergency contraception) was a statistically significant predictor of correctly answering all questions except condom usage. For example, compared to women who have not had a Pap smear in the last year, those who had a Pap smear had a 1.71 greater odds answering all Pap smear questions correctly.

**Table 4:** Univariable logistic regression analyses

	**AGE**			**RACIAL/ETHNIC GROUP**			**INSURANCE**	**HEALTH HISTORY**		
	*OR (95% CIs)*	*OR (95% CIs)*	*OR (95% CIs)*	*OR (95% CIs)*	*OR (95% CIs)*	*OR (95% CIs)*	*OR (95% CIs)*	*OR (95% CIs)*		

**PAP SMEARS**										
	**26–35**	**36–45**	**46–55**	**Other**	**Black**	**Hispanic**	**Private**	**Pap < 1 year**		
**Who**	1.64 (1.03–2.48)	1.53 (1.00–2.36)	2.14 (1.23–3.72)	0.36 (0.22–0.58)	0.38 (0.23–0.64)	0.24 (0.16–0.38)	2.57 (1.82–3.61)	1.46 (1.03–2.06)		
**Why**	1.80 (1.19–2.73)	1.99 (1.28–3.10)	3.05 (1.68–5.51)	0.47 (0.29–0.76)	0.47 (0.28–0.78)	0.36 (0.23–0.56)	3.40 (2.38–4.86)	1.54 (1.09–2.18)		
**When**	1.72 (1.13–2.61)	1.98 (1.26–3.11)	2.61 (1.46–4.68)	0.28 (0.17–0.46)	0.25 (0.15–0.41)	0.42 (0.26–0.68)	3.07 (2.15–4.39)	1.41 (0.99–2.01)		
**All correct**	1.85 (1.27–2.71)	2.33 (1.56–3.48)	3.41 (2.08–5.60)	0.28 (0.18–0.46)	0.31 (0.19–0.51)	0.27 (0.17–0.43)	3.14 (2.31–4.27)	1.71 (1.24–2.36)		
**BREAST SELF-EXAMS**										
	**26–35**	**36–45**	**46–55**	**Other**	**Black**	**Hispanic**	**Private**	**BSE < 1 month**		
**Who**	0.90 (0.45–1.78)	0.76 (0.38–1.51)	0.86 (0.38–1.97)	0.72 (0.31–1.63)	0.74 (0.31–1.77)	0.33 (0.18–0.63)	3.66 (2.01–6.67)	2.00 (1.43–2.79)		
**Why**	0.74 (0.32–1.71)	0.57 (0.25–1.30)	0.91 (0.31–2.68)	0.42 (0.17–1.07)	0.53 (0.19–1.51)	0.28 (0.13–0.62)	3.23 (1.55–6.74)	1.09 (0.72–1.63)		
**When**	1.36 (0.85–2.18)	1.94 (1.13–3.32)	1.97 (1.02–3.79)	0.50 (0.28–0.87)	0.45 (0.25–0.80)	0.41 (0.24–0.68)	2.61 (1.74–3.93)	2.03 (1.59–2.60)		
**All correct**	1.45 (0.92–2.28)	1.57 (0.97–2.55)	1.45 (0.82–2.55)	0.64 (0.37–1.09)	0.55 (0.32–0.95)	0.39 (0.24–0.62)	2.66 (1.82–3.87)	1.95 (1.56–2.46)		
**MAMMOGRAMS**										
	**26–35**	**36–45**	**46–55**	**Other**	**Black**	**Hispanic**	**Private**	**Mammo < 1 year**		
**Who**	2.15 (1.47–3.15)	2.19 (1.47–3.27)	2.08 (1.30–3.31)	0.52 (0.33–0.82)	0.67 (0.42–1.08)	0.56 (0.37–0.86)	2.72 (1.76–4.20)	0.96 (0.67–1.37)		
**Why**	2.46 (1.38–4.38)	1.90 (1.07–3.35)	2.88 (1.31–6.32)	0.36 (0.20–0.65)	0.60 (0.29–1.24)	0.34 (0.19–0.60)	3.66 (2.01–6.66)	0.71 (0.40–1.29)		
**When**	1.39 (0.95–2.02)	2.54 (1.67–3.88)	4.63 (2.63–8.16)	0.37 (0.23–0.58)	0.59 (0.36–0.96)	0.44 (0.29–0.67)	3.59 (2.33–5.54)	0.37 (0.25–0.57)		
**All correct**	1.68 (1.12–2.53)	2.21 (1.45–3.37)	2.54 (1.56–4.12)	0.36 (0.21–0.61)	0.58 (0.35–0.97)	0.38 (0.23–0.61)	3.68 (2.21–6.11)	0.60 (0.42–0.86)		
**BIRTH CONTROL PILLS**										
	**26–35**	**36–45**	**46–55**	**Other**	**Black**	**Hispanic**	**Private**	**BCP users (cur.)**		
**Who**	0.49 (0.26–0.92)	0.28 (0.15–5.53)	0.51 (0.24–1.08)	0.41 (0.23–0.75)	0.46 (0.24–0.88)	0.72 (0.38–1.38)	2.75 (1.71–4.44)	0.29 (0.13–0.69)		
**Why**	0.19 (0.05–0.69)	0.19 (0.05–0.71)	0.30 (0.07–1.38)	0.45 (0.16–1.23)	0.78 (0.22–2.84)	0.63 (0.22–1.83)	4.83 (1.81–12.91)	infinite		
**When**	0.61 (0.30–1.24)	0.69 (0.32–1.47)	0.75 (0.31–1.85)	0.60 (0.27–1.33)	0.50 (0.22–1.11)	0.46 (0.23–0.94)	2.28 (1.27–4.09)	0.31 (0.11–0.86)		
**All correct**	0.64 (0.38–1.09)	0.45 (0.27–0.77)	0.57 (0.31–1.06)	0.45 (0.26–0.78)	0.47 (0.27–0.84)	0.65 (0.38–1.14)	2.74 (1.81–4.16)	0.27 (0.13–0.57)		
**EMERGENCY CONTRACEPTION**										
	**26–35**	**36–45**	**46–55**	**Other**	**Black**	**Hispanic**	**Private**	**EC users**		
**EC possible**	0.62 (0.40–0.96)	0.76 (0.48–1.19)	0.74 (0.43–1.26)	0.89 (0.53–1.51)	1.41 (0.84–2.36)	1.03 (0.63–1.66)	1.59 (1.13–2.24)	0.38 (0.22–0.64)		
**CONDOMS**								**Condom use**		
	**26–35**	**36–45**	**46–55**	**Other**	**Black**	**Hispanic**	**Private**	**Sometimes**	**Often**	**Always**
**Who**	0.62 (0.30–1.30)	0.56 (0.26–1.18)	0.61 (0.26–1.46)	0.67 (0.30–1.47)	1.36 (0.46–4.01)	0.56 (0.27–1.14)	3.16 (1.68–5.93)	1.70 (0.72–3.97)	1.92 (0.72–5.08)	2.17 (0.82–5.73)
**Why**	0.33 (0.08–1.30)	0.21 (0.06–0.80)	0.24 (0.06–1.01)	0.30 (0.10–0.89)	0.52 (0.13–1.99)	0.41 (0.13–1.28)	2.45 (1.00–6.04)	1.16 (0.37–3.68)	1.88 (0.41–8.53)	2.09 (0.46–9.50)
**When**	0.77 (0.34–1.75)	0.35 (0.17–0.74)	1.67(0.46–6.06)	0.44 (0.19–1.02)	0.85 (0.28–2.57)	0.29 (0.14–.59)	2.37 (1.26–4.47)	1.08 (0.49–2.38)	1.10 (0.46–2.64)	3.01 (0.89–10.17)
**All correct**	0.60 (0.32–1.09)	0.43 (0.24–0.79)	0.73 (0.33–1.58)	0.68 (0.35–1.33)	1.29 (0.53–3.16)	0.42 (0.24–0.74)	2.12 (1.31–3.42)	1.09 (0.58–2.05)	1.68 (0.76–3.71)	1.78 (0.72–4.41)
REFERENCE GROUPS							KEY TO ABREVIATIONS			
**Age: **18–25	**Health History**	**Pap: **Pap Smear > 1 year		**BC Users: **Non-current BCP Users						
**Racial/Ethnic Group: **White		**BSE: **BSE > 1 month		**EC Users: **Never used EC			PAP: Papanicolaou	BCP: Birth Control Pills		
**Insurance: **Non-private		**Mammogram: **Mammogram > 1 year		**Condom Use: **Never use condoms			BSE: Breast Self Exam	EC: Emergency Contraception		

Table 5 displays the results of the multivariable logistic regression analyses. In these analyses, age group, racial/ethnic group, having private medical insurance, and current, recent or ever (for emergency contraception only) use of the cancer screening or contraceptive methods were used as covariates in the models. In the regression models employing the composite outcome of correctly answering all questions on a given topic, older age remained associated with greater Pap smear and mammogram knowledge; being of white race was associated with greater Pap smear knowledge and was generally associated with greater mammogram knowledge; being Hispanic/Latina was associated with lesser Pap smear, breast self-exam, mammogram, and condom knowledge; having private medical insurance was associated with greater knowledge for all six topics; and recent or current usage of a given screening or contraceptive method was associated with greater Pap smear, breast self-examination, and birth control pill knowledge, and ever use of emergency contraception was associated with greater emergency contraception knowledge.

**Table 5:** Multivariable logistic regression analyses

		**AGE**			**RACIAL/ETHNIC GROUP**			**INSURANCE**	**HEALTH HISTORY**		
	***n***	*OR (95% CIs)*	*OR (95% CIs)*	*OR (95% CIs)*	*OR (95% CIs)*	*OR (95% CIs)*	*OR (95% CIs)*	*OR (95% CIs)*	*OR (95% CIs)*		
**PAP SMEARS**											
		**26–35**	**36–45**	**46–55**	**Other**	**Black**	**Hispanic**	**Private ins**	**Pap < 1 year**		
**Who**	702	1.70 (1.09–2.66)	1.44 (0.90–2.30)	2.01 (1.09–3.70)	0.47 (0.28–0.78)	0.42 (0.24–0.71)	0.30 (0.19–0.49)	1.91 (1.32–2.76)	1.51 (1.04–2.21)		
**Why**	699	1.88 (1.21–2.92)	2.12 (1.30–3.44)	2.96 (1.56–5.62)	0.68 (0.40–1.14)	0.54 (0.32–0.94)	0.53 (0.33–0.87)	2.99 (2.04–4.37)	1.67 (1.14–2.44)		
**When**	698	1.88 (1.20–2.95)	2.18 (1.33–3.59)	2.95 (1.52–5.71)	0.38 (0.23–0.64)	0.29 (0.17–0.49)	0.60 (0.36–1.00)	2.32 (1.57–3.42)	1.46 (0.98–2.16)		
**All correct**	694	2.11 (1.40–3.18)	2.53 (1.63–3.94)	3.77 (2.16–6.57)	0.39 (0.23–0.64)	0.34 (0.20–0.57)	0.37 (0.23–0.61)	2.35 (1.68–3.29)	1.83 (1.27–2.62)		
**BREAST SELF-EXAMS**											
		**26–35**	**36–45**	**46–55**	**Other**	**Black**	**Hispanic**	**Private ins**	**BSE < 1 month**		
**Who**	702	0.84 (0.41–1.70)	0.63 (0.29–1.35)	0.57 (0.23–1.43)	1.12 (0.45–2.77)	0.91 (0.37–2.24)	0.48 (0.24–0.96)	3.32 (1.72–6.42)	1.91 (1.34–2.74)		
**Why**	702	0.66 (0.27–1.58)	0.53 (0.21–1.34)	0.77 (0.23–2.60)	0.60 (0.22–1.67)	0.61 (0.21–1.81)	0.32 (0.14–0.76)	2.76 (1.24–6.16)	1.07 (0.69–1.66)		
**When**	699	1.26 (0.76–3.44)	1.28 (0.64–2.56)	0.56 (0.31–1.03)	0.56 (0.31–1.03)	0.50 (0.27–0.93)	0.61 (0.35–1.06)	2.07 (1.32–3.23)	1.90 (1.46–2.47)		
**All correct**	698	1.37 (0.85–2.21)	1.31 (0.78–2.23)	0.99 (0.54–1.83)	0.81 (0.45–1.45)	0.63 (0.35–1.13)	0.51 (0.31–0.85)	2.28 (1.51–3.43)	1.89 (1.48–2.41)		
**MAMMOGRAMS**											
		**26–35**	**36–45**	**46–55**	**Other**	**Black**	**Hispanic**	**Private ins**	**Mammo < 1 year**		
**Who**	677	2.18 (1.47–3.25)	2.32 (1.49–3.63)	2.16 (1.23–3.77)	0.67 (0.41–1.09)	0.79 (0.48–1.33)	0.75 (0.47–1.18)	1.84 (1.33–2.56)	1.42 (0.92–2.20)		
**Why**	678	2.25 (1.23–4.14)	2.13 (1.06–4.31)	2.58 (0.99–6.74)	0.43 (0.22–0.84)	0.67 (0.31–1.44)	0.48 (0.25–0.91)	2.68 (1.56–4.61)	1.22 (0.59–2.51)		
**When**	674	1.41 (0.94–2.11)	2.58 (1.59–4.19)	3.42 (1.77–6.64)	0.45 (0.27–0.75)	0.68 (0.39–1.17)	0.61 (0.38–0.98)	2.22 (1.57–3.14)	0.71 (0.43–1.18)		
**All correct**	673	1.77 (1.15–2.71)	2.10 (1.31.3.36)	1.88 (1.05–3.37)	0.53 (0.31–0.92)	0.69 (0.40–1.19)	0.51 (0.30–0.85)	2.21 (1.56–3.12)	0.88 (0.56–1.37)		
**BIRTH CONTROL PILLS**											
		**26–35**	**36–45**	**46–55**	**Other**	**Black**	**Hispanic**	**Private ins**	**BC users**		
**Who**	694	0.48 (0.25–0.94)	0.33 (0.17–0.63)	0.55 (0.24–1.26)	0.55 (0.29–1.06)	0.53 (0.27–1.04)	0.84 (0.41–1.69)	2.49 (1.47–4.20)	0.52 (0.21–1.30)		
**Why**	569	0.20 (0.06–0.75)	0.36 (0.09–1.48)	0.28 (0.06–1.30)	0.67 (0.23–1.93)	0.95 (0.25–3.60)	0.84 (0.27–2.62)	5.08 (1.66–15.57)	infinity		
**When**	696	0.64 (0.31–1.31)	0.75 (0.34–1.67)	0.75 (0.30–1.88)	0.77 (0.34–1.77)	0.58 (0.25–1.33)	0.54 (0.26–1.14)	1.90 (1.03–3.53)	0.41 (0.14–1.19)		
**All correct**	692	0.66 (0.38–1.13)	0.51 (0.29–0.90)	0.60 (0.31–1.18)	0.62 (0.34–1.10)	0.56 (0.31–1.03)	0.74 (1.52–3.73)	2.38 (1.52–3.73)	0.42 (0.19–0.91)		
**EMERGENCY CONTRACEPTION**											
		**26–35**	**36–45**	**46–55**	**Other**	**Black**	**Hispanic**	**Private ins**	**EC users**		
**EC possible**	688	0.61 (0.39–0.96)	0.76 (0.48–1.20)	0.69 (0.40–1.19)	1.08 (0.62–1.88)	1.63 (0.94–2.81)	1.20 (0.72–2.01)	1.78 (1.22–2.59)	0.36 (0.21–0.63)		
**CONDOMS**									**Condom use**		
		**26–35**	**36–45**	**46–55**	**Other**	**Black**	**Hispanic**	**Private ins**	**Sometimes**	**Often**	**Always**
**Who**	629	0.74 (0.32–1.73)	0.78 (0.30–2.02)	0.47 (0.17–1.30)	0.70 (0.29–1.71)	1.76 (0.50–6.22)	0.76 (0.32–1.82)	3.23 (1.52–6.85)	1.62 (0.63–4.17)	1.43 (0.51–4.01)	1.71 (0.62–4.72)
**Why**	628	0.26 (0.05–1.36)	0.22 (0.04–1.19)	0.14 (0.02–0.84)	0.22 (0.06–0.84)	0.34 (0.08–1.52)	0.34 (0.08–1.40)	1.95 (0.64–5.95)	0.72 (0.21–2.53)	0.91 (0.18–4.67)	1.31 (0.27–6.41)
**When**	627	0.83 (0.33–2.08)	0.30 (0.12–0.72)	1.01 (0.25–4.00)	0.49 (0.20–1.25)	1.32 (0.48–3.59)	0.41 (0.21–0.80)	1.94 (1.11–3.39)	1.07 (0.54–2.11)	1.25 (0.54–2.90)	1.53 (0.67–3.50)
**All correct**	624	0.67 (0.34–1.34)	0.41 (0.20–0.86)	0.63 (0.26–1.56)	0.63 (0.30–1.32)	1.32 (0.48–3.59)	0.41 (0.21–0.80)	1.94 (1.11–3.39)	1.07 (0.54–2.11)	1.25 (0.54–2.90)	1.53 (0.67–3.50)
REFERENCE GROUPS								KEY TO ABREVIATIONS			
**Age: **18–25		**Health History**	**Pap: **Pap Smear > 1 year		**BC Users: **Non-current BCP Users						
**Racial/Ethnic Group: **White			**BSE: **BSE > 1 month		**EC Users: **Never used EC			PAP: Papanicolaou		BCP: Birth Control Pills	
**Insurance: **Non-private			**Mammogram: **Mammogram > 1 year		**Condom Use: **Never use condoms			BSE: Breast Self Exam		EC: Emergency Contraception	

## Discussion

For these 18–55-year-old women visiting the ED, we observed several interesting patterns of women's cancer screening and contraceptive knowledge. We noted areas in which knowledge of the applicability, purpose, and recommended usage intervals of these six cancer screening and contraceptive modalities was strong or poor, as well as groups with greater or lower knowledge levels. These findings should help the health care providers identify patients who might require more extensive discussions to evaluate the patient's prior usage of these methods. The findings also provide a cautionary note to researchers and clinicians who intend to assess the need for preventive health services for women in EDs. Female ED patients who do not have private health care insurance, those who do not use these methods, and perhaps Hispanic/Latina patients might not understand the applicability, purpose, and recommended intervals of these six cancer screening and contraceptive methods, and therefore might inaccurately relate their need for or prior use of them. Further, these women might benefit from expanded discussions with ED providers in initiatives that try to increase patient utilization of these methods.

Knowledge about the six women's cancer screening and contraceptive methods varied across topics, and for some methods, knowledge varied within topics. For example, knowledge about the purpose and applicability of breast self-examinations was high; however, fewer women knew the recommended intervals. Knowledge of who may take birth control pills was lower than why and how often they should be taken. Perhaps recent press regarding efforts to produce a male birth control pill may have confused participants. Lower knowledge of how often women should undergo mammograms and who should get them may be a consequence of conflicting messages from public health groups (and perhaps health care providers) on this subject. Knowledge of emergency contraception was quite poor, a finding that is supported elsewhere [12,13,16]. The findings from this study demonstrate that knowledge across and within these topics is variable and helps to identify areas of reduced knowledge. This variability needs to be accounted for when assessing patient understanding of various women's cancer screening and contraceptive methods and in the planning of interventions to increase usage of these methods.

Two factors, having private medical insurance and recent or current usage of certain women's cancer screening and contraceptive methods, were statistically significant predictors of knowledge in the multivariable logistic regression models. Patients who had governmental (Medicare, Medicaid, or both) medical insurance and patients who did not have any type of insurance demonstrated lesser cancer screening and contraceptive knowledge compared to those with private medical insurance. Our findings are supported by results from other studies [14,17-23,27,29]. For example, in Takakuwa et al.'s study, having private medical insurance was positively associated with greater knowledge of preventive behaviors regarding breast cancer [29], and in Han et al.'s study, women who had never received a clinical breast exam exhibited significantly lower knowledge of clinical breast exams [18]. Knowledge was not perfectly associated with consistent usage, however. Most women understood well the who, when, and how often condoms should be used – although few used them consistently.

It is likely that having private medical insurance reflects higher socioeconomic status, which is associated with greater educational opportunities and enhanced contact with medical providers. Although not addressed in this study, other studies have shown that having a primary care or obstetrician/gynecologist provider and having recently visited one is associated with greater knowledge of screening and contraceptive methods [16,24] and usage of these methods [45-47]. Also not examined in this study, other studies have reported greater knowledge about cancer screening and contraception among women with more years of formal education [14,15,21,22,25-27]. It is possible that private medical insurance might be a rough proxy for having or visiting an obstetrician/gynecologist or primary care provider and receiving information about cancer screening or contraceptive methods. However, we cannot determine from the results of this study if women with private medical insurance are receiving more comprehensive health care education from their medical providers or from other sources. It is probably true that health care education from whatever source and usage synergistically contributes to greater knowledge and utilization. Women who were using or had recently used screening and contraceptive methods generally answered more questions correctly than other women. However, we cannot determine causal or temporal relationships from this survey. Women with a greater knowledge of preventive health may undergo screening more frequently, or women undergoing screening may learn more about the purpose and recommended intervals of screening through the screening process. Nevertheless, the association of having private medical insurance and more recent or ever usage of screening and contraceptive methods observed here suggests that women without private medical insurance or who have not recently undergone screening may benefit from expanded discussions with their ED providers when assessing their need for these cancer screening and contraceptive methods.

To our surprise, age and race/ethnicity were less consistently associated with women's preventive health cancer screening and contraceptive knowledge than anticipated. We expected that age would be a strongly associated factor with knowledge since age reflects information gathered over repeated exposure to screening, life experiences, and educational opportunities. Age has been associated with women's cancer screening and contraceptive knowledge in other studies [9,11,22,26,27]. There was a trend of lesser knowledge among the Hispanic/Latina participants in the survey. Other studies have observed reduced cancer screening or contraception knowledge among Hispanics/Latinas [22,23,27,29]. However, we anticipated that race/ethnicity, which is associated with access to medical care, socioeconomic status, and educational levels, to be strongly associated with preventive health care knowledge as it was in other studies [29,30,48]. For example, ethnic background was associated with knowledge and understanding of breast-self exams, clinical breast exams and mammograms in a 2004 study of ethno-cultural groups in Ontario [30]. Byrd et al. noted similar findings in a study of cervical cancer screening among Hispanic women [48]. In a study by Takakuwa et al. white and Native American respondents exhibited greater knowledge concerning the frequency of breast self-exams than among African American, Asians, or Hispanics [29]. It is possible that having private medical insurance, which is a strong marker of access to health care, may better account for knowledge of women's cancer screening and contraception than race/ethnicity or age.

There are several limitations of this investigation. The study was from a single ED and involved English-speaking women predominately from certain demographic groups (e.g. white, Catholic, single, privately insured), so the study findings may not be applicable to other settings and populations. Given our sampling techniques, we believe that we have a representative sample of those eligible to participate in the study. This fact combined with our relatively large sample suggests that our observations, in general, are valid. We are hopeful that our survey will be adapted for other settings, cultures, and languages to corroborate our results. It is also possible that, despite our pilot study involving cognitive assessments and screening of questions, the participants did not understand certain questions, did not accurately recall their health history, or were afraid to answer some questions. Inaccurate recall or misunderstanding of their health history, such as incorrectly stating the time since the last Pap smear, might lead to incorrect estimates of the relationship between usage and knowledge. As we conjecture in this study, accurate estimates of usage are likely affected by patient knowledge. Since we did not review the patients' medical records, we cannot assess the extent of this problem. It is reassuring that few patients did not participate because of the nature of the questions. We were unable to assess age-specific relationships in this study, e.g., age greater than 40 and knowledge of mammograms, because we asked patients to select their age group instead of state their age to enhance truthfulness in responses to this question.

## Conclusion

Although ED clinicians frequently ask female patients about their usage of screening and contraceptive methods, it is clear that patient knowledge within and across these topics is variable and is sometimes incomplete. Women who do not have private medical insurance and those who have not recently or currently used a women's cancer screening or contraceptive method have lesser knowledge about them. ED clinicians should take into account the potential for knowledge deficits about women's cancer screening and contraceptive knowledge when obtaining health histories. Future assessments that examine the need for women's cancer screening and contraceptive methods among ED patients and proposed interventions to increase usage should take account the variability of patient knowledge on these topics.

## Abbreviations

ED: emergency department

Pap: Papanicolaou

## Competing interests

The author(s) declare that they have no competing interests.

## Authors' contributions

RCM and EMG were the primary authors of the survey and participated in all phases of the study, its analysis, and manuscript preparation. BCB assisted with the construction of the survey, analysis, and manuscript preparation. BMB and MAC were involved with the study analysis and manuscript preparation. All authors read and approved the final manuscript.

## Pre-publication history

The pre-publication history for this paper can be accessed here:


